# Predictive Value of Gait Speed and Grip Strength for Mortality in Older Adults with Cardiometabolic Multimorbidity

**DOI:** 10.1093/geroni/igaf122.4178

**Published:** 2025-12-31

**Authors:** Hyeon Hong, Shweta Gore, Lin-Na Chou, Julie Keysor, Amol Karmarkar, Amit Kumar

**Affiliations:** University of Utah, Salt Lake City, Utah, United States; MGH Institute of Health Professions, Boston, Massachusetts, United States; University of Utah, Saratoga Springs, Utah, United States; University of Massachusetts Lowell, Lowell, Massachusetts, United States; Virginia Commonwealth University, Richmond, Virginia, United States; University of Utah, Salt Lake City, Utah, United States

## Abstract

This study aims to establish cut-off points for gait speed and hand grip strength based on one-year hospitalization risks and to examine their predictive value for three-year mortality in older adults with cardiometabolic multimorbidity (CMM). This retrospective cohort study utilized data from the National Health and Aging Trends Study (2015-2018) linked to Medicare claims for community-dwelling adults aged 66 years and older. Our primary predictors were gait speed (m/s) and BMI-normalized hand-grip strength. First, we established optimal cut-off values for gait speed and grip strength based on 1-year hospitalization risk, using sensitivity and specificity. Next, we employed Cox proportional hazards regression to estimate the probability of 3-year mortality, with gait speed and grip strength as predictors, stratified by sex and disease group. Among 4,157 older adults,11.3% had CMM (6.5% male, 4.7% female). Among older males, those with CMM had the lowest cut-off values for grip strength at 0.82kg/BMI (sensitivity 0.59; specificity 0.85) and for gait speed at 0.58m/sec (sensitivity 0.62; specificity 0.92) to predict 1-year hospitalization. In females with CMM, the cut-off was 0.52kg/BMI (sensitivity 0.61; specificity 0.84) for grip strength and 0.68m/sec (sensitivity 0.69; specificity0.71) for gait speed. Among males with CMM, those with below the cut-off gait speed had a significantly higher 3-year mortality compared to those with above the cut-off (HR = 0.47, 95% CI = 0.23-0.97, p < 0.05). These validated cut-off points for gait speed and grip strength can assist in risk stratification and mortality prediction in older adults with CMM, serving as a screening tool for identifying high-risk individuals.

